# The protective effect of 3% diquafosol on meibomian gland morphology in glaucoma patients treated with prostaglandin analogs: a 12-month follow-up study

**DOI:** 10.1186/s12886-020-01550-6

**Published:** 2020-07-10

**Authors:** Yue Guo, Jun Young Ha, He Long Piao, Mi Sun Sung, Sang Woo Park

**Affiliations:** 1 Department of Ophthalmology and Research Institute of Medical Sciences, Chonnam National University Medical School and Hospital, 42 Jebong-ro, Dong-Gu, Gwangju, 61469 South Korea; 2 Eye Hospital and School of Ophthalmology and Optometry, Wenzhou Medical University, Wenzhou, Zhejiang, China; 3 Department of Ophthalmology, Yanbian University Hospital, Yanji, Jilin, China

**Keywords:** Diquafosol, glaucoma, Prostaglandin analogs, Meibomian gland morphology, Preservatives

## Abstract

**Background:**

To determine if 3% diquafosol (DQS) can preserve the meibomian gland morphology in glaucoma patients treated with prostaglandin analogs (PGA) for a 12-month follow-up period.

**Methods:**

This study included 84 eyes of 46 normal tension glaucoma (NTG) patients who were treated with either preservative-containing PGA (PC-PGA; 16 patients, 28 eyes), preservative-free PGA (PF-PGA; 21 patients, 39 eyes), or a combination of PC-PGA and 3% DQS (PC-PGA + DQS; 9 patients, 17 eyes). The meibography of the upper eyelid was acquired using Keratograph® 5 M at baseline and at each follow-up (1, 3, 6, 9, and 12 months). Meibomian gland loss (MGL) was quantitatively analyzed by using ImageJ software.

**Results:**

In the PC-PGA group, MGL increased significantly from baseline to month 9 and month 12, whereas no significant changes were observed in the PF-PGA and PC-PGA + DQS groups during the entire 12 months. All groups showed similar MGL at each follow-up time from baseline to six months. However, MGL in the PC-PGA group was significantly higher than those in the PF-PGA and PC-PGA + DQS groups at the 9 and 12 months.

**Conclusions:**

Combining 3% DQS with PC-PGA was as effective as PF-PGA in preserving the meibomian gland morphology for at least 12 months. Our results suggest that 3% DQS may be a promising strategy for managing glaucoma patients with a high risk of developing meibomian gland dysfunction due to preservative-containing topical medications.

## Background

The treatment of glaucoma usually starts with topical anti-glaucoma medications, of which prostaglandin analogs (PGA) have been recommended as the first-line choice because of their robust intraocular pressure (IOP) lowering effect, well-tolerated systemic profile, and convenient once-daily dosing [1]. However, adverse ocular events such as conjunctival hyperemia, eyelash elongation, and iris pigmentation might occur after immediate or long-term use of PGA [2, 3]. Recently, a high prevalence of meibomian gland dysfunction (MGD) was observed in patients treated with PGA [4–6]. MGD is a distinct disease characterized by terminal duct obstruction and/or qualitative/quantitative changes in glandular secretion, which may result in alterations of the tear film, symptoms of eye irritation, clinically apparent inflammation, and ocular surface disease [7]. Furthermore, we found an association between MGD and the compliance of preservative-containing PGA (PC-PGA) in patients with normal tension glaucoma (NTG) [8]. Collectively, MGD may exacerbate the ocular subjective symptoms and compromise medication compliance, leading to unstable IOP control and glaucoma progression eventually. Therefore, it is of great importance to preserve the meibomian gland while using PGA for glaucoma management.

It was speculated that both active ingredients and preservatives contained in PGA might contribute to MGD through direct toxicity or chronic inflammation [4–6]. Preservative-free PGA (PF-PGA) was developed to eliminate the detrimental impact of preservatives on the ocular surface. Several studies showed that PF-PGA caused less damage to the meibomian gland than the corresponding PC-PGA [9–12]. However, the initial status of the meibomian gland and the effects of PGA over time were not determined because of the cross-sectional design approach used in previous studies. Moreover, not all PGA are available in the preservative-free formulations that limits the use of PF-PGA among glaucoma patients with MGD. Thus, other strategies need to be considered to alleviate MGD for PGA users.

Diquafosol (DQS), a P2Y2 receptor agonist, is currently recognized as an emerging topical medication for dry eye therapy because it promotes tear fluid and mucin secretion [13]. Notably, the P2Y2 receptors were found not only in the corneal and conjunctival epithelium but also in the meibomian gland, which suggests a potential role of DQS in stimulating the function of the meibomian gland [14, 15]. Several clinical trials also demonstrated that DQS could alleviate MGD-related signs and symptoms [16–20]. Nevertheless, no definitive evidence has been provided on its effectiveness in protecting the meibomian gland of PGA users.

Hence, the purpose of this study was to determine whether 3% DQS is beneficial to the meibomian gland of PC-PGA users by investigating the changes in the meibomian gland morphology over a 12-month period and comparing with those who only used PC-PGA or PF-PGA.

## Methods

### Subjects

Patients attending the glaucoma clinic in the Department of Ophthalmology (Chonnam National University Hospital) with features of NTG were consecutively enrolled in this prospective study between September 2018 and February 2019. This study adhered to the Declaration of Helsinki and was approved by the Chonnam National University Hospital Institutional Review Board. Written informed consents were obtained after the subjects were fully informed of the purposes of this study.

NTG was diagnosed using the following criteria: untreated maximum IOP lower than 21 mmHg during the repeated Goldmann applanation tonometry measurements taken in a routine period of daytime on different days, a normal open angle observed on gonioscopy, and the occurrence of glaucomatous optic neuropathy identified in fundus photographs (Kowa Nonmyd 7 fundus camera; Kowa Co., Ltd., Tokyo, Japan) and optical coherence tomography (OCT) (Cirrus HD-OCT; Carl Zeiss Meditec Inc., Dublin, CA, USA) with the corresponding visual field (VF) defects assessed using automated perimetry (Humphrey Field Analyzer; Carl Zeiss Meditec Inc., Dublin, CA, USA). A glaucomatous VF defect was defined as a cluster of three or more contiguous points in the pattern deviation plot with *P* < 0.05, at least one of which must have been *P* < 0.01; a pattern standard deviation with *P* < 0.05; or a glaucoma hemifield test result outside of normal limits. VF defects had to be repeatable on at least 2 subsequent tests with reliable analyses (that is, false-positive rate of ≤15%, false-negative rate of ≤15%, and fixation loss rate of < 20%).

To qualify for inclusion, the subjects were required to satisfy the following criteria: 18 years or older with newly diagnosed NTG, starting one kind of PGA treatment within two weeks before enrollment, and no MGD-related signs, including abnormal lid margin, altered gland secretions, or conjunctival and corneal staining [21]. Patients were excluded if they had blepharitis, wore contact lens, used other topical eye drops except for PGA or DQS, had undergone ocular surgery, had androgen deficiency, rosacea, or Stevens-Johnson syndrome, or were undergoing systemic medication treatment (such as isotretinoin, antiandrogens, antidepressants, antihistamines or postmenopausal hormone therapy). These are all factors that may affect the meibomian gland [22].

According to the topical medications used by patients, they were classified into the PC-PGA monotherapy group, PF-PGA monotherapy group, and PC-PGA with 3% DQS (PC-PGA + DQS) combination therapy group. PC-PGA included latanoprost 0.005% with benzalkonium chloride (BAK) 0.02% (Xalatan®, Pfizer Inc., New York, USA) and tafluprost 0.0015% with BAK 0.001% (Taflotan®, Santen Pharmaceutical Co, Ltd., Osaka, Japan). PF-PGA included latanoprost 0.005% without BAK (Monoprost®, Thea, Clermont-Ferrand, France) and tafluprost 0.0015% without BAK (Taflotan-S®, Santen Pharmaceutical Co, Ltd., Osaka, Japan). Three percent DQS ophthalmic solution without BAK (Diquas-S®; Santen, Osaka, Japan) was originally intended for those who were worried about dry eye symptoms. All patients were instructed by the glaucoma specialists to apply PGA once per night and DQS four times per day (if appropriate) before enrollment.

Apart from the routine glaucoma examinations which involve the assessment of the IOP, optic nerve head, retinal nerve fiber layer thickness, and VF, the meibomian gland morphology evaluation was the main focus of this study. All examinations were performed at baseline and at each follow-up (1, 3, 6, 9, and 12 months).

### Meibomian gland morphology evaluation

To evaluate the meibomian gland morphology, non-contact infrared meibography was performed for the upper eyelid of each eye using Keratograph® 5 M (OCULUS, Wetzlar, Germany) [23]. Photographs from the meibography were then analyzed by using ImageJ 1.52a (National Institute of Health, USA) to calculate the ratio of the meibomian gland dropout area to the total tarsal area. This ratio was named the meibomian gland loss (MGL; %), which was found to be reliable for grading the meibomian gland morphology [24, 25].

**Fig. 1:**
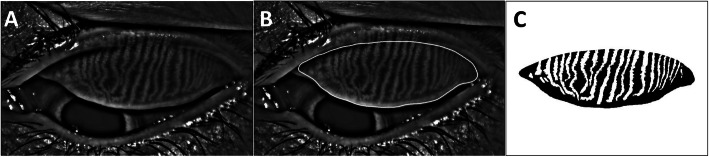
Meibography image analyzed using ImageJ. A, Original image. B, The total tarsal area was encircled with a solid white line. C, Automatic threshold identification was applied. White and black denote the meibomian gland area and the meibomian gland dropout area, respectively

More specifically, we first manually outlined the total tarsal area using the polygon selection tool. Next, the original color photograph was converted into an 8-bit type image. We then applied automatic threshold identification to discriminate the meibomian gland area from the non-meibomian gland area [26]. The non-meibomian gland area was regarded as the meibomian gland dropout area. In case the automatic threshold identification was not performed properly, the threshold was manually adjusted and the misidentified area was modified using the paintbrush tool. Finally, the number of pixels within the meibomian gland dropout area was counted and its relation to the pixels in the total tarsal area was calculated as a fraction (0–100%) (Fig. 1).

MGL of each meibography photograph was evaluated by two experienced observers (Y.G. and J.Y.H.) who were masked to the treatment of each patient and the results of prior observations. The mean MGL value was analyzed statistically.

### Statistical analysis

Statistical analysis was performed with the SPSS software (version 21.0, SPSS, Inc., Chicago, IL, USA). The normality of distribution was verified using the Kolmogorov–Smirnov test. Differences in the demographics among groups were compared using one-way analysis of variance (ANOVA). Categorical variables were compared using the chi-square test. The inter-observer agreement for the MGL measurement was evaluated by calculating the intraclass correlation coefficients. The assumption of sphericity was verified using the Mauchly’s test. In case the data violated the sphericity assumption, Greenhous–Geisser correction was applied. Repeated measures ANOVA with post hoc Bonferroni correction was used to compare MGL at different follow-up time points of each group. Multivariate ANOVA with post hoc Bonferroni correction was used to compare MGL among different groups at each follow-up time point. All data were expressed as mean ± standard deviation unless otherwise indicated. *P* < 0.05 was considered statistically significant.

## Results

Initially, 93 eyes from 51 NTG patients were enrolled. After assessment for eligibility, 5 patients were excluded due to violation of the inclusion criteria or unwillingness to participate. As a result, a total of 46 NTG patients (84 eyes) were finally included in this study. Among them 16 patients (28 eyes) were treated with PC-PGA, 21 patients (39 eyes) were treated with PF-PGA, and 9 patients (17 eyes) were treated with PC-PGA and 3% DQS. Demographic characteristics are summarized in Table 1. As indicated, there were no significant differences in age (*P* = 0.5), gender (*P* = 0.272), IOP (*P* = 0.528), and type of ingredient in PGA (*P* = 0.853) among the three groups.

**Table 1:** Demographics of Participants

	PC-PGA (16 subjects, 28 eyes)	PF-PGA (21 subjects, 39 eyes)	PC-PGA + DQS (9 subjects, 17 eyes)	***P***
Age (y)	63.9 ± 11.6	60.2 ± 15.4	63.9 ± 10.1	0.500^a^
Sex (male/female)	6/10	9/12	3/6	0.272^b^
IOP (mmHg)	15.8 ± 3.4	16.4 ± 1.8	16.7 ± 2.4	0.528^a^
Type of ingredient (Latanoprost/Tafluprost)	14/14	19/20	8/9	0.853^b^

Values are represented as mean ± SD

ANOVA = analysis of variance; DQS = diquafosol; IOP = intraocular pressure; PC-PGA = preservative-containing prostaglandin analogs; PC-PGA + DQS = preservative-containing prostaglandin analogs and diquafosol; PF-PGA = preservative-free prostaglandin analogs; SD = standard deviation

^a^One-way ANOVA test

^b^Chi-square test

Compared with baseline, IOP was reduced in all three groups at 12 months (15.8 ± 3.4 mmHg vs 13.5 ± 2.1 mmHg, 16.4 ± 1.8 mmHg vs 14.6 ± 1.7 mmHg, and 16.7 ± 2.4 mmHg vs 13.9 ± 2.3 mmHg for PC-PGA, PF-PGA, and PC-PGA + DQS group, respectively). There was no significant difference in IOP among the three groups at 12 months (*P* = 0.077). In the PC-PGA group, 6 patients (37.5%) complained about slight ocular dryness and irritation after 9 months of treatment, whereas no subjective symptoms were reported in the PF-PGA and PC-PGA + DQS groups throughout the whole follow-up period. Moreover, no significant ocular surface complications were noted in the three groups after treatment.

MGL measurement showed excellent inter-observer reproducibility with an intraclass correlation coefficient of 0.869 (range: 0.737–0.937, *P* < 0.001).

**Fig. 2:**
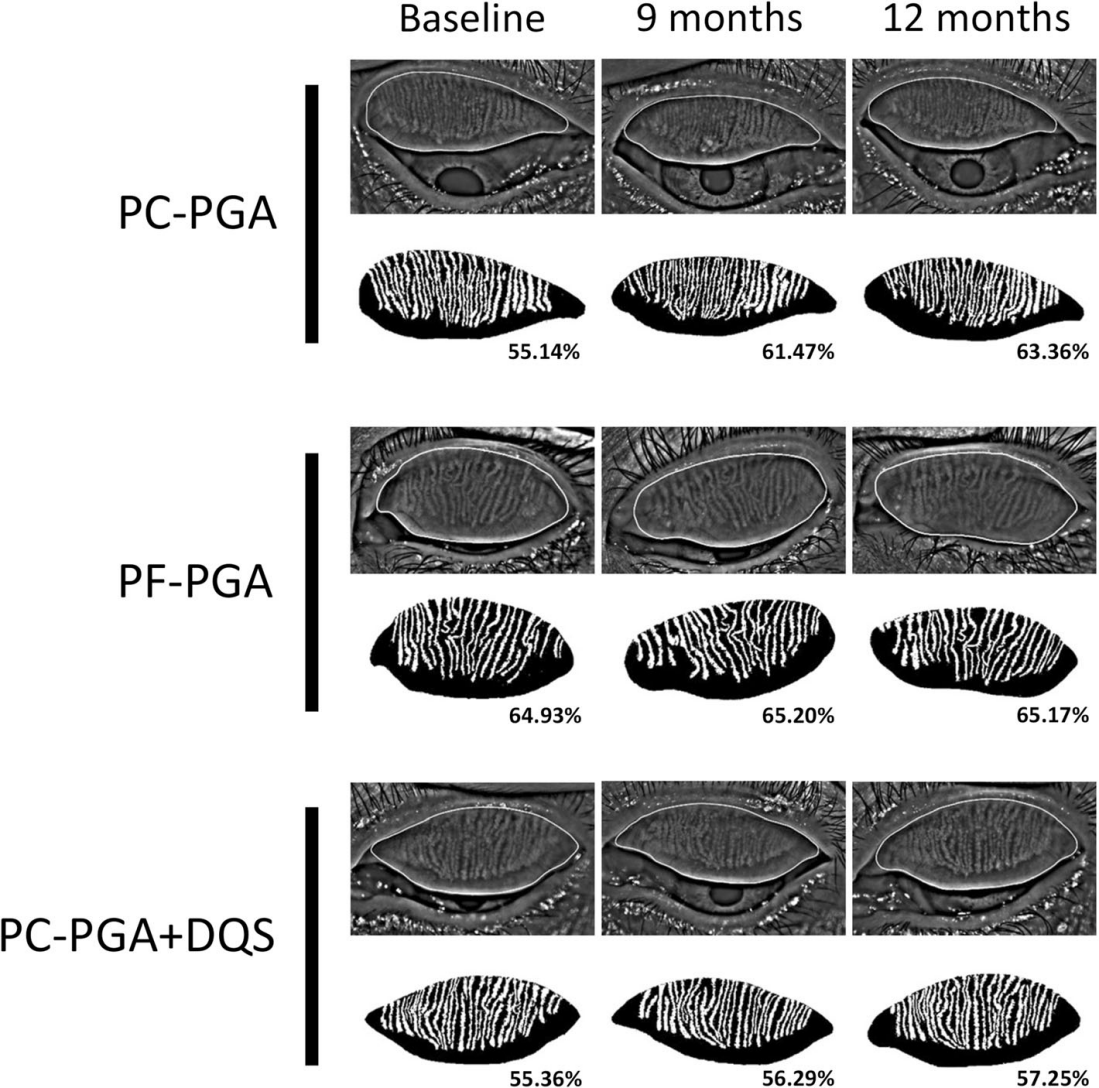
Representative meibography images of three NTG patients. MGL of a 57-year-old woman significantly increased from 55.14% at baseline to 61.47% at the 9 months and 63.36% at the 12 months after PC-PGA treatment (upper two panels). No obvious changes in MGL were observed in an 80-year-old man treated with PF-PGA (middle two panels) and a 60-year-old woman treated with PC-PGA + DQS (bottom two panels). DQS = diquafosol; MGL = meibomian gland loss; NTG = normal tension glaucoma; PC-PGA = preservative-containing prostaglandin analogs; PC-PGA + DQS = preservative-containing prostaglandin analogs and diquafosol; PF-PGA = preservative-free prostaglandin analogs

**Fig. 3:**
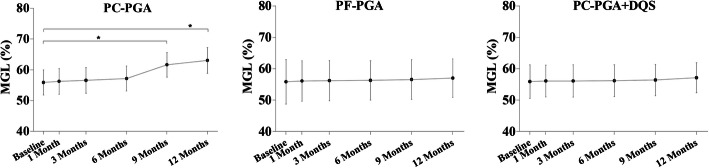
Graph showing variations in MGL following PC-PGA, PF-PGA, and PC-PGA + DQS treatment. *P < 0.001, repeated measures ANOVA with post hoc Bonferroni correction. ANOVA = analysis of variance; DQS = diquafosol; MGL = meibomian gland loss; PC-PGA = preservative-containing prostaglandin analogs; PC-PGA + DQS = preservative-containing prostaglandin analogs and diquafosol; PF-PGA = preservative-free prostaglandin analogs

Compared with baseline, MGL was significantly increased at the 9 and 12 months in the PC-PGA group (all *P* < 0.001), whereas no significant changes were found in the PF-PGA and PC-PGA + DQS groups (all *P* > 0.05; Table 2, Fig. 2). As illustrated in Fig. 3, MGL increased with time in the PC-PGA group but remained stable in the PF-PGA and PC-PGA + DQS groups for 12 months.

**Table 2:** MGL of Each Group over 12-month Period

Group	n (eyes)	MGL (%) at different follow-up time					
		Baseline	1 month	3 months	6 months	9 months	12 months
PC-PGA	28	55.89 ± 4.05	56.21 ± 4.15	56.55 ± 4.16	57.14 ± 4.03	61.59 ± 4.00^a^	63.03 ± 4.18^a^
PF-PGA	39	55.86 ± 7.07	56.10 ± 6.46	56.22 ± 6.38	56.30 ± 6.24	56.55 ± 6.29^b^	57.02 ± 6.15^b^
PC-PGA + DQS	17	55.91 ± 5.36	56.11 ± 5.07	56.12 ± 5.12	56.20 ± 5.04	56.42 ± 4.95^b^	57.16 ± 4.78^b^

Values are represented as mean ± SD

ANOVA = analysis of variance; DQS = diquafosol; IOP = intraocular pressure; MGL = meibomian gland loss; PC-PGA = preservative-containing prostaglandin analogs; PC-PGA + DQS = preservative-containing prostaglandin analogs and diquafosol; PF-PGA = preservative-free prostaglandin analogs; SD = standard deviation

^a^Repeated measures ANOVA with post hoc Bonferroni correction, *P* < 0.001, vs baseline in the same group

^b^Multivariate ANOVA with post hoc Bonferroni correction, *P* < 0.01, vs PC-PGA group at the same follow-up time

**Fig. 4:**
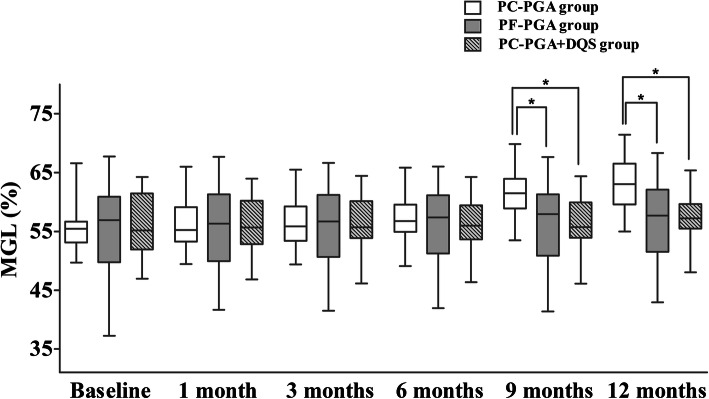
Comparison of MGL among the PC-PGA, PF-PGA, and PC-PGA + DQS groups. Box plots indicate the 25th to 75th percentile, and the 50th percentile (the median) is denoted by a horizontal line in the box. The whiskers at the top and bottom of the box plots indicate the maximum and minimum values, respectively. *P < 0.01, multivariate ANOVA with post hoc Bonferroni correction. ANOVA = analysis of variance; DQS = diquafosol; MGL = meibomian gland loss; PC-PGA = preservative-containing prostaglandin analogs; PC-PGA + DQS = preservative-containing prostaglandin analogs and diquafosol; PF-PGA = preservative-free prostaglandin analogs

All groups showed similar MGL at each follow-up time from baseline to six months (all *P* > 0.05). However, MGL in the PC-PGA group was significantly higher than those in the PF-PGA and PC-PGA + DQS groups at the 9 and 12 months (all *P* < 0.01; Table 2, Fig. 4).

## Discussion

Current perspectives on the medical treatment of glaucoma focus not only on effectiveness but also on safety and compliance. Previous studies reported significant anomalies of the meibomian gland in PGA users [4–6]. We also found an association between MGD and the reduced compliance of PGA in our previous study [8]. Given the high frequency of MGD among PGA users and the underlying adverse effect on treatment, it is important to protect the meibomian gland during PGA treatment.

The present study showed that the MGL values for all groups were similar at baseline. However, MGL of the PC-PGA group increased gradually during treatment, whereas those of the other two groups remained stable. The results indicate that PC-PGA negatively affects the meibomian gland morphology in a time-dependent manner, possibly because of the cumulative effects of the active ingredients or preservatives. Considering the MGL of PF-PGA users did not show significant changes, we speculate that the preservative is the main contributor to the deterioration of meibomian gland morphology. This is in accordance with a previous in vitro study, in which the preservative was found to be cytotoxic to human meibomian gland epithelial cells and might act synergistically with PGA leading to higher toxicity [12]. Furthermore, in vivo confocal microscopy studies suggested that the preservative might exaggerate the effect of PGA by facilitating the penetration of PGA into the meibomian gland [9–11]. Therefore, removing preservatives from PGA is an effective way to maintain the meibomian gland morphology.

To our knowledge, only one clinical study has investigated the effect of DQS in glaucoma patients. In that study, DQS was effective in improving dry eye-related signs and symptoms with no impact on IOP [27]. However, no information regarding its effect on the meibomian gland was provided. Our present study supports their findings since no significant difference in IOP was detected among the three treatment groups at 12 months, indicating that DQS has no effect on IOP. Moreover, our study sheds some light on the protective effect of DQS on the meibomian gland morphology in PGA users, even though the precise mechanism underlying this protective effect is still unclear. A potential explanation may be that DQS can be absorbed by palpebral conjunctiva and acts on the P2Y2 receptors present in the meibomian gland to promote lipid secretion. The release of lipid subsequently eases the pressure inside the gland duct and acinus, thus alleviating gland atrophy. In addition, DQS is known to promote tear fluid secretion. Sufficient tears may attenuate the inflammation caused by the PC-PGA which would be beneficial to postpone the progression of gland atrophy.

In this study, we used MGL to quantitatively evaluate the meibomian gland morphology. It is because the most common morphological changes of meibomian glands such as gland shortening, distortion, and dropout would result in decreased meibomian gland area, namely increased non-meibomian gland area. Therefore, the ratio of non-meibomian gland area to the total tarsal area could represent the meibomian gland morphology. According to the previous studies, objectively assessing the area of meibomian gland loss was useful for evaluating the subtle morphological changes of meibomian glands [28, 29]. In addition, MGL evaluation using ImageJ was found to be more sensitive and reliable than evaluation using the subjective grading scale [24, 25]. We only assessed MGL in the upper eyelids as the lower eyelids in our patients were too tight to be completely everted and non-fully everted eyelid would interfere with the quality of the meibography. Dogan et al. [30] also suggested that the upper lid might be the preferred lid for the evaluation of meibomian gland dropout.

This study had some limitations. First, the group size was relatively small. Second, we mainly focused on the morphology of the meibomian gland but not on the other ocular surface parameters. To fully understand the effect of DQS will aid to guide the treatment of PGA-related ocular surface diseases in addition to MGD. Third, the duration of observation was only 12 months. MGL may progress after the long-term use of PC-PGA even when it is combined with DQS. Further studies are warranted to evaluate the effect of DQS on both meibomian gland and other ocular surface parameters in patients who use PGA over a long-term.

## Conclusions

In conclusion, PC-PGA induced a significant loss of the meibomian gland in a time-dependent manner. In contrast, there were no significant changes in MGL in the PF-PGA group. These findings indicate that the preservative might play a predominant role in the development of MGL. Importantly, combining 3% DQS with PC-PGA was as effective as PF-PGA in protecting the morphologic integrity of the meibomian gland for at least 12 months. From these findings, we suggest that 3% DQS may be a promising therapeutic agent for alleviating MGD in glaucoma patients treated with preservative-containing topical medications.

## Data Availability

The datasets analyzed in this study are available from the corresponding author (Sang Woo Park, exo70@naver.com) upon reasonable request.

## Abbreviations

ANOVA: Analysis of variance
BAK: Benzalkonium chloride
DQS: Diquafosol
IOP: Intraocular pressure
MGL: Meibomian gland loss
NTG: Normal tension glaucoma
OCT: Optical coherence tomography
PGA: Prostaglandin analogs
PC-PGA: Preservative-containing prostaglandin analogs
PC-PGA + DQS: Preservative-containing prostaglandin analogs and diquafosol
PF-PGA: Preservative-free prostaglandin analogs
VF: Visual field
